# Community perceived barriers to uptake of health services among men at Sedibeng district in South Africa

**DOI:** 10.4102/hsag.v29i0.2548

**Published:** 2024-04-15

**Authors:** Ndumiso Tshuma, Daniel N. Elakpa, Clinton Moyo, Tshepo M. Ndhlovu, Mathildah M. Mokgatle, Sangiwe Moyo, Sehlule Moyo, Martha Chadyiwa, Mandeep K. Kochar, Mokgadi Malahlela, Takalani G. Tshitangano, David D. Mphuthi

**Affiliations:** 1 The Best Health Solutions, Johannesburg, South Africa; 2 Center for Health Policy, University of the Witwatersrand, Johannesburg, South Africa; 3 Department of Public Health, School of Healthcare Sciences, Sefako Makgatho Health Sciences University, Pretoria, South Africa; 4 The Final Mile, Johannesburg, South Africa; 5 Department of Environmental Health, University of Johannesburg, Johannesburg, South Africa; 6 Bombay Teachers’ Training College, HSNC University, Colaba, Mumbai, India; 7 Department of Public Health, University of Venda, Limpopo, South Africa; 8 Department of Health Studies, University of South Africa, Johannesburg, South Africa

**Keywords:** health-services, men, South Africa, access barriers, Facebook

## Abstract

**Background:**

This qualitative study aimed to investigate the barriers that hinder men’s utilisation of healthcare services in the Sedibeng district of South Africa.

**Methods:**

The study was conducted using flyers with questions posted on the Best Health Solutions’ Facebook page for two weeks. A convenience sampling method was used and a total of 104 comments were collected from 64 respondents. The authors analysed the participants’ self-reported data thematically on demographics, geographic area, and reasons for men not using healthcare services.

**Results:**

The findings revealed that sociocultural norms played a significant role in men’s reluctance to seek medical help, as it was perceived as a sign of weakness. The scarcity of male healthcare professionals hinders open discussions. Stigma and discrimination were identified as substantial barriers. Convenience, trust, and confidentiality concerns, along with the influence of intimate partners, also influence men’s decision-making.

**Conclusion:**

This study highlights the complex interplay between various barriers that impact men’s utilisation of healthcare services in the district. By addressing these factors, healthcare providers and policymakers can enhance healthcare access and promote better health outcomes for men in Sedibeng district.

**Contribution:**

The manuscript’s primary contribution lies in uncovering multifaceted barriers to men’s healthcare utilisation in Sedibeng district. It explores socio-cultural norms, healthcare worker demographics, stigma, discrimination, convenience factors, trust, confidentiality concerns, and the influence of intimate partners on men’s healthcare choices. These insights illuminate the complex factors affecting men’s healthcare access, providing valuable knowledge for healthcare providers and policymakers.

## Introduction

According to a recent report by the World Health Organization (WHO), the average global life expectancy for males is 70.9 years, while females have a life expectancy of 75.9 years (WHO 2021). This indicates that women tend to live approximately 5 years longer than men do on a global scale. Although men often enjoy greater opportunities, privileges and societal power than women in many communities, this advantage does not necessarily translate into better health outcomes (Baker et al. 2014). It is estimated that more than 52% of all disease-related deaths among men can be prevented globally (Etienne 2018). For human immunodeficiency virus (HIV) and acquired immunodeficiency syndrome (AIDS), men are tested, initiated and retained in HIV treatment less than women, contributing to the growing life expectancy gap between men and women (Hlongwa et al. 2022). As elsewhere in the world, men in South Africa seek medical help at a later stage than women and receive more informal care (Mbokazi et al. 2020). In South Africa, 57% of all tuberculosis (TB) deaths occur in men, and men aged 45 years and older account for most deaths from non-communicable diseases (NCD), such as diabetes and cerebrovascular disease (NDoH 2017).

Although extensive research has explored the relationship between access to healthcare between men and women (Crimmins et al. 2019), few studies have focused on the reasons why men choose not to visit clinics (Alcalde-Rubio et al. 2020). The continued increase in studies pointing to gender disparities in mortality rate conveys a need for research that extends beyond statistical knowledge to qualitative research grounded in understanding men’s lives, behaviours and many choices beyond those offered by the health systems. This need is illustrated in many ways; for example, Sedibeng is substantially better supplied with healthcare services, yet there is still a low uptake of healthcare services among men when compared to other districts in the country.

It is increasingly evident that a low uptake of health services will continue to contribute to a high mortality rate among men; thus, it is critical to identify and address the barriers. Understanding the different barriers and their contribution to men’s limited access to health services is an expected criterion for sustainable intervention. For this reason, the authors leveraged ‘Facebook’ to reach out to the communities in the Sedibeng district to understand the challenges that men face in accessing healthcare services.

However, because of the emergence of e-epidemiology, under which Facebook falls, the scope expanded to include all individuals in the Sedibeng district who interacted with our post. As noted by Nelson et al. (2019), e-epidemiology offers possibilities for reaching understudied populations and conducting large-scale studies. However, the respondents provided a reasonable profile of key individuals involved in the key decision-making processes of men in their access to healthcare services. They included intimate partners, healthcare workers, personnel from community-based organisations, and most importantly, men.

Sedibeng district consists of more people above the age of 40 than the South African average but has a significantly lower number of men above the age of 70 when compared to women (Coorperate Governance and Traditional Affairs 2020). In this study, the authors assessed the barriers to the uptake of healthcare services among men in the Sedibeng district in South Africa. Our motivation for undertaking this study is to investigate the factors contributing to barriers in the utilisation of health services among men in the Sedibeng district of South Africa. There is a dearth of research on this specific aspect in Sedibeng district, and this could be attributed to a predominant historical emphasis on broader health disparities, with a particular focus on women and children (Bertakis et al. 2000; Hunt et al. 2011). In response to this research gap, our study aims to provide a nuanced understanding of the challenges faced by men in accessing health services in the Sedibeng district.

## Research methods and design

### Research design

This study was rooted in a qualitative study design, which describes human experiences based on the perspectives provided by the participants. The purpose of using this study design was to allow participants to describe from their experience the obstacles that confront men in seeking and accessing healthcare services without the interference of the research team or anyone else. The phenomenological approach is highly recommended for understanding complex health stories in human-centred design. Facebook allowed respondents to freely express their experiences without facing time constraints or interruptions from the researchers or other participants, as is usually the case with focus group meetings and individual interviews.

### Study setting

This study was conducted on the Best Health Solutions’ Facebook page where a flyer was posted. Facebook plays a crucial role in disseminating health information and engaging with communities from a health perspective (The Best Health Solutions 2020). It serves as a platform for health education, offering accurate and timely information through articles, videos and live sessions (The Best Health Solutions 2020). Communities and support groups on Facebook enable individuals to connect, seek advice and provide emotional support for various health challenges. The Best Health Solutions runs health awareness campaigns, showcase programme work and gather data on her Facebook platform.

The research was conducted on The Best Health Solutions, where a flyer was posted. Facebook plays a pivotal role in the dissemination of health-related information and community engagement with a specific health focus, as highlighted in this systematic review of social media use for health purposes (Chen & Wang 2021). Social media platforms serve as an invaluable conduit for health education, delivering timely, reliable information through diverse formats, including articles, videos and live sessions. Furthermore, Facebook’s community and support groups empower individuals to connect, exchange advice and offer emotional support to those grappling with a range of health problems. The Best Health Solutions actively employs their Facebook page to conduct health awareness campaigns, showcase their programme initiatives, and collect essential information.

### Study conceptualisation

**FIGURE 1:**
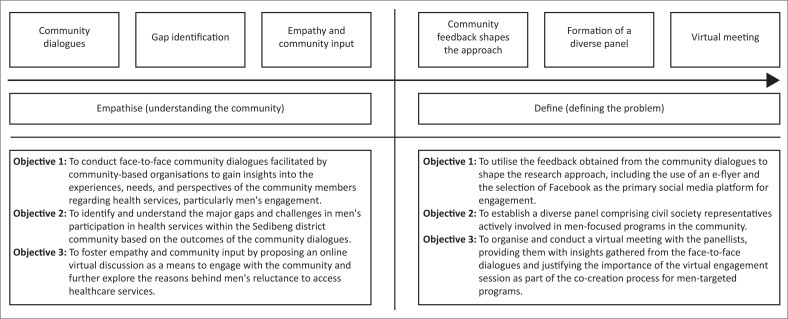
Flow diagram illustrating the conceptualisation of the project.

In the initial phase of conceptualising this study, the authors adopted a human-centred design approach, which aligns with the ‘Empathise’ and ‘Define’ stages of problem-solving. This approach emphasises understanding the community’s needs, concerns and perspectives as a foundation for developing effective solutions. Our journey towards investigating the barriers to health service uptake among men in the Sedibeng district, South Africa began with a deep and empathetic understanding of the community (Figure 1).

#### Step 1: Community dialogues

The study’s inception was influenced by two face-to-face community dialogues with a total of 51 participants (13 men), facilitated by two community-based organisations operating in the Sedibeng district. These organisations were supported by Best Health Solutions and Networking HIV and AIDS Community of Southern Africa (NACOSA) both of which are non-profit entities committed to assisting community-based organisations address community health issues. The two dialogues provided a platform for community members to openly share their thoughts, concerns and experiences related to health services and what must be prioritised.

#### Step 2: Identification of a gap

The community members who participated in the community dialogues identified and prioritised men’s health-seeking behaviour and accessing health services. It became evident that men were notably absent from active participation in health services and were reluctant to seek necessary healthcare. This recognition served as the starting point for our study.

#### Step 3: Empathy and community input

The two facilitators who led the face-to-face dialogues were acutely aware of the need for more inclusive and comprehensive male engagement in healthcare. To address this gap, a novel idea was proposed – an online virtual discussion. The topic of this discussion centred on the question: ‘What makes men not go to clinics in Sedibeng district: How can we make men go to clinics to access health and gender-based violence-related services?’ Importantly, the proposed discussion format would allow for a wider audience reach and inclusivity.

#### Step 4: Community feedback shapes the approach

The participants in these face-to-face dialogues played an essential role in shaping the research approach. They suggested using an e-flyer and specifically endorsed the use of Facebook as the primary social media platform for this engagement, citing its popularity and accessibility in the community.

#### Step 5: Formation of a diverse panel

Community members, during the dialogues, proposed the formation of a diverse panel comprising eight civil society representatives actively engaged in programmes focusing on men’s health and well-being. Additionally, the authors purposively selected four additional panellists from community-based organisations involved in men-focused programmes in Gauteng, Limpopo and North West provinces.

#### Step 6: Virtual meeting

All panellists attended a virtual meeting where they were briefed on the outcomes of the face-to-face dialogues. This meeting aimed to present the justification for holding a virtual engagement session as a crucial component of the co-creation process for men-targeted programmes. The collaboration between community voices and civil society representatives in this meeting was essential in defining the next steps of the project.

### Sampling, data collection and analysis

**FIGURE 2:**
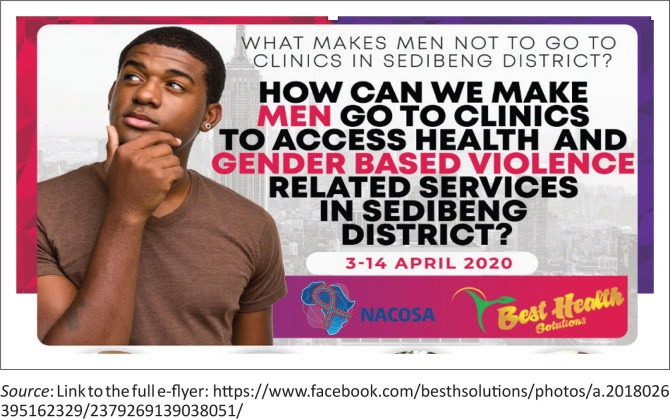
The design of the e-flyer.

A convenience sampling method was used, and the e-flyer was initially published on the Best Health Solutions (Figure 2), and the link was subsequently shared within various WhatsApp groups and featured on the WhatsApp status of the panellists. During the period from 03 April 2020 to 14 April 2020, panellists actively engaged with the community, liking comments and prompting respondents. The research team performed the data collection through extraction of comments from Facebook, which was then meticulously reviewed and coded to ensure data reliability and trustworthiness. Addressing any disparities in the inter-coder reliability process, the authors iteratively refined codes and themes in alignment with existing literature. The MAXQDA qualitative software was used for analysis. Thematic content analysis was employed to analyse the data, involving a thorough inductive coding process of all comments for the identification and consolidation of meaningful units into emerging categories and themes. The analysis and coding process was spearheaded by DE, NT, TN, and CM.

### Ethical considerations

Ethical approval was obtained from University of Johannesburg Research Ethics Committee, Faculty of Health Sciences with the approval number REC-1541-2022, NHREC Registration: REC 241112-035 for the Process Evaluation of the Community Response System in South Africa. During the workshop, participants were briefed regarding the data collection process for the purposes of the research study, and they were assured that every identifying information would be removed. Recognising the potential sensitivity of sharing responses publicly, participants were offered the alternative option of privately submitting their contributions via a designated chat box. After the data-collection period, the post was archived.

## Results

A total of 104 comments were posted publicly and 11 sent privately to the Best Health Solutions Facebook Messenger chat. A total of 64 respondents participated over the period of 2 weeks; the gender distribution was 23 females and 41 males. From our results, the structure of the health sector and cultural reasons were the main factors affecting men’s access to healthcare services. Respondents emphasised that there is a shortage of male nurses in South Africa, which affects the demand for healthcare services among men. This demand for male nurses is considered a derived demand for health services and is rooted in an innate sociocultural construct. However, internal mental models, alternative medicine, intimate partners, and convenience reasons also largely contribute to men’s uptake of biomedical and health services.

### Structural barriers

The authors found that access to healthcare services among men is not the only convenience problem. Instead, the authors found that the structure of the healthcare sector has an overarching effect on the demand for healthcare services, particularly in public hospitals and clinics situated within communities. The structural factors which comprise characteristics such as the socio-demographic characteristics of the health workforce, location and economic factors play an important role. Furthermore, the intersection of the participants’ cultural beliefs and the structure of the healthcare sector further exacerbates negative attitudes towards clinics and hospitals among men. A male participant suggested that more male nurses are needed in the health system because of the broader socio-ecosystem that governs patterns of relations between men and women. He said he would be more comfortable talking about his sexual reproductive health to a male nurse.

### Shortage of male nurses in the healthcare sector

Some men admitted that the high presence of female nurses and the subsequent shortage of male nurses were two of the factors affecting their demand for health services. Public clinics, which became a huge form of reference as a barrier among men, were met with negative action tendencies because of the high presence of women. Participants suggested that in Africa, gender is the main arranging principle of power, and that men find it difficult to give away that power by communicating their physical pain to female nurses. Some male participants registered displeasure owing to the lack of respect exhibited by female nurses in their contexts. The high prevalence of female nurses in clinics upsets social inequality by serving as a disadvantage that shapes men’s negative attitudes towards clinics. They reiterated that communicating their pain to female nurses is accompanied by a loss of comfort and seemingly uneasy conversation because it threatens their engrained belief that they are superior to women, as illustrated by the participants’ comments:

‘Yes, there is a massive shortage. In general, us African men we are reluctant to engage in key topics by their female counterparts. When dealing with a female, we do not really open up to a level where we can adequately lay out our true feelings and perspectives. There is also a general perception that female health practitioners are disrespectful and tend to belittle people. A man is reluctant to engage with a person who is not trustworthy.’ (Male)

Another male participant stated that being examined by a woman is a huge concern for him because it is something that men are not used to:

‘The healthcare system needs to enrol more male nurses. Domination of the health system by females contributes to men’s perceived resistance. For example, I can talk about my manhood issues better to a male health worker than a female health worker because of my upbringing. I was raised to talk to my dad about my manhood issues not my mom.’ (Male)

A female nurse indicated and confirmed that men did not want to be served by a woman:

‘They [*men*] do not want to be serviced by women as much as they believe that no woman must be involved in men’s issues. They are also afraid of getting tested for HIV.’ (Female nurse)

Another male respondent reiterated that female nurses are rude and judgemental towards men, making clinics an uncomfortable place for men:

‘Additionally, there is a perception that female nurses in public clinics are rude and judgemental towards men. Current nurses and healthcare providers could be trained to communicate more effectively with male patients in a sensitive, non-judgemental way and to develop a rapport prior to a physical exam. It is also possible that having some male sexual health care providers, in addition to female providers, may aid in making clinics a more comfortable place for men.’ (Male)

### Stigma and discrimination

The results demonstrated that men’s refusal to seek healthcare services is exacerbated by multiple forms of stigma, discrimination and other human rights violations that occur as a result of antiretroviral pick-up points and gossip within communities. In particular, respondents indicated that some health facilities located within the Sedibeng district carry the stigma of HIV.

A female nurse noted that antiretroviral therapy (ART) pickup points are known in the community, thus making men shy away from accessing and receiving ART services and medications as well as other sexual reproductive health services:

‘As has been shown in the Sedibeng district, some of the things that lead to men not going to the health centres are the stigma attached to each facility. Most people get discriminated against because their partner is taking certain medications, which leads to gossip in small cities and towns.’ (Female nurse)

Another male participant said that a mixture of long queues, a high presence of women, and stigma drives men away from seeking healthcare services from clinics in the district:

‘Men always think the clinic is for women; some of us think people will talk about us if seen visiting the clinic, we rather do traditional, some are disappointed by long queues, and end up not going to clinic.’ (Male)

### Culture and upbringing as a barrier

The responses also indicate that men’s health decisions are based on dominant notions and practices that prevail in cultural contexts and upbringing. The participants indicated that men resisted attending clinics because they were taught to hide any signs of weakness. The core barrier of culture and upbringing fuels the denial of illness.

### Denialism

Female respondents purported that their intimate partners dismissed falling sick as a passing event or over-inflated threat. They proposed that upbringing underlies denialism and leads to rationalising non-adherence to seeking help from medical facilities because falling sick is taken as a weakness. Some men reported that they ran away from clinics to avoid the possibility of testing positive for critical illness.

One female respondent suggested that men tend to be well until they are too late:

‘Men comprehend that a body is like a car that needs to be a service. They were brought up in believing that sickness is associated with being weak and they never acknowledge when they are not well or when they need help and this result in them presenting for medical care when it’s too late to intervene.’ (Female)

Another female respondent suggested that men are socially conditioned not to seek help:

‘Unfortunately, men are taught from an early age, either by cultural referencing or by direct parenting, to be tough, not to cry, or to show any form of emotion as it is seen as a weakness. It’s been instilled in them to “cocoon” everything up unable to open up even to their partners let alone to a stranger at a health care facility, so how then do we expect them to be emotionally intelligent enough to open up when they need help, yet we expect them to “never” need help.’ (Female)

A female nurse linked men’s denial with compulsory HIV testing, arguing that men pretended to be tough and hoped that any sickness would heal miraculously:

‘I think men always pretend to be strong, seek help is a sign of weakness, and are scared of compulsory HIV testing. Others think that if they are sick, whatever they feel will miraculously disappear.’ (Female)

One male participant pointed out that society expects men to not complain or ask for help; hence, seeking medical help is seen as a weakness:

‘I do not know how this belief began. Men perceive attending health facilities as a weakness. This is similar to the inability to handle one’s issues. We wish not to complain or ask for any help. We sort things out of ourselves. When all else fails.’ (Male)

### Anticipated negative consequences of testing positive for illness

Some men reported that not going to clinics was perpetuated by the inability to cope with an unexpected diagnosis of a critical illness. They expressed concerns about not being able to handle positive test results.

Knowing one’s positive status of an illness is considered a burden, as noted by one of the male participants:

‘Some men believe that if you go to a clinic, doctors will find an illness that will make you lose your peace of mind. And yet if you do not, you will never know and be happy.’ (Male)

### Intimate partners as a barrier

Some female participants submitted that they shared their medications with their partners out of sympathy. This also enables men not to seek help from health facilities:

‘Naturally, as women are loving and caring when my male partner is sick, I would share my MEDS with him, any meds.’ (Female)

Another participant reiterated that sexual partners provided an enabling environment for men not to test for HIV. He posits that when their partners test negative, they also assume that they are HIV negative:

‘The patriarchal system that men grew affected current men as they were taught not to cry, not to go to clinics, and when they are ill, they should not show. Hence, you find them testing with their partners thinking when the partners are automatically negative. Therefore the need for behavioural change programmes.’ (Male)

### Unaffordable men’s clinics

The data collected show that male clinics provide a better environment for men to seek and access healthcare services because of the presence of male nurses and high levels of respect given to clients. However, the upward development of male clinics in South Africa was established through the private sector and remains unaffordable and inaccessible to many men.

One participant proposed that the privatisation of men’s clinics makes them inaccessible to men:

‘Most of the clinic’s 90% health workers are females and men are not at ease with discussing the health problems with females; I think men’s clinics should be in our general health facilities not private and be accessible.’ (Female)

A transgender participant indicated that improving the attitude of female nurses would help create an environmentally friendly place for men to access health facilities:

‘There is a need to improve health workers’ attitudes in local clinics, and imagine what will happen if a man is succumbed to that?.’ (Transgender)

### Alternative medicine

Traditional herbs are a preferred alternative in clinics because of their high levels of confidentiality, lack of stigma and discrimination, and easy access. The presence of alternatives drives men away from clinics and hospitals:

‘Whenever I get sick, I just drink my concoctions. It always helps strengthen my immune system and clean my blood. African medicine always helps; I do not see the need for a hospital.’ (Male)

### Lack of education

Finally, participants suggested that a lack of sexual reproductive health education leads to unfavourable decision-making processes towards seeking help about sexually transmitted infections among men and boys:

‘The subject of Sexual Reproductive Health is not well taught in our communities and schools; hence, most people are scared or feel shy and do not want to be examined, especially with issues related to sexually transmitted infections. This subject requires more attention, especially in young and adolescent boys. When people understand their sexuality, they also understand the importance of looking after themselves. Therefore, it is necessary to strengthen this subject.’ (Male)

## Discussion

In this study, the authors aimed to assess the barriers to the uptake of healthcare services among men in the Sedibeng district of South Africa. By adopting this human-centred approach, the authors have laid a strong foundation for our project. The ‘Empathise’ and ‘Define’ phases have equipped us with a comprehensive understanding of the community’s needs and concerns, and the insights gathered have shaped the trajectory of this research towards innovative and community-driven solutions for improving men’s participation and vulnerability to seek health care in the Sedibeng district. The subsequent stages, ‘Ideate’ and ‘Prototype’, will build upon this empathetic foundation as the authors work towards creating effective interventions.

Our results showed that the major barriers were the demography of health workers, sociocultural issues, stigma and discrimination. Other identified factors were the cost of healthcare, use of traditional medicines, convenience, lack of health education, fear of disease outcomes, having multiple intimate partners and an internal mental model of men.

### Cultural and social factors

Men’s willingness to seek healthcare services can be influenced by sociocultural norms. In some cultures, seeking medical help is considered a sign of weakness or vulnerability (Lemos et al. 2017). This agrees with our findings that says ‘…men often perceive seeking medical care as a sign of weakness due to societal expectations and upbringing…’, ‘…they (men) tend to suppress emotions and believe that they should handle health issues on their own…’, ‘…this mindset leads to delayed medical intervention and a reluctance to ask for help, potentially resulting in more serious health conditions…’. This leads to denial and hiding of symptoms of ailments. Another study revealed that participants (men) ignored symptoms because of feelings of vulnerability and embarrassment. They constructed a perception of men as strong and self-reliant, while women on the one hand were more proactive in seeking assistance; on the other hand, men were described as requiring prompting to address their health issues (Jeffries & Grogan 2012).

### Demography of health workers and gender-specific health issues

This is a significant barrier to the uptake of health services by men. Our results found that, in some cases, men might prefer to seek healthcare services from providers who have a better understanding of their sex-specific health issues. Male respondents reported that they felt more comfortable discussing sensitive topics such as prostate or sexual and reproductive health issues with male healthcare professionals. This perception as a barrier to accessing health services may be reinforced if most of the healthcare workforce consists of women. Evidence of this phenomenon is consistent with a study conducted in New Zealand, where they found that the demography of the health workforce affects the decision to access certain types of services among most men (McKinlay, Kljakovic & McBain 2009; Tudiver & Talbot 1999). The failure to cope with receiving health services from female nurses reflects how much culture and upbringing influence men’s health-seeking behaviour. Thus, if the health workforce lacks adequate representation of male practitioners in these areas, it may deter them from accessing such services. Flexible restructuring of the healthcare sector should meet the specific cultural needs of the people. Moreso, training, and recruitment initiatives must focus on proactively attracting and encouraging nurses to be respectful and exercise patience with patients.

Achieving the right staffing personnel of committed and competent PHC structures is critical to ensuring the delivery of healthcare objectives to the South African population.

As a branching logic of demographic barriers, the distribution of the health workforce in areas such as age, ethnicity and cultural background can influence the level of comfort and trust men feel during their interactions with healthcare providers. Men may feel more at ease discussing their health concerns with healthcare professionals, who they perceive as relatable, respectful or share similar experiences (Ab Rahman, Al-Sadat & Yun Low 2011; Jeffries & Grogan 2012). In doing so, they feel safe from embarrassment, discrimination and stigma. The feeling of being warmly welcomed into a health service centre can promote trust between patients and providers, which may improve patients’ vulnerability. Our results showed that female health practitioners who form a larger proportion of the workforce are perceived as disrespectful, belittling, rude and judgemental towards men. This contributes to stigma and discrimination, and as such, creates a negative environment in clinics, making them uncomfortable and unwelcoming for men. Similarly, previous studies have found that men highlight the shortcomings of healthcare services, which include inadequate availability, inconvenience and an unwelcoming atmosphere. They expressed the need to be greeted warmly and establish a personal connection with healthcare providers (Coles et al. 2010; Silva et al. 2016).

More importantly, the results indicate that we should consider a range of contextual factors that can influence men’s decision to access healthcare services, such as privacy and confidentiality.

Our results indicate that gossip and discrimination were linked to partners taking certain medications, further perpetuating stigma within small cities and towns. This speaks greatly about the lack of trust and confidentiality in health service delivery in these settings. Trust and confidentiality are essential elements in patient–provider relationships and play a significant role in patients’ engagement with healthcare services and better outcomes (Birkhäuer et al. 2017). Men are naturally reluctant to engage with health practitioners and do not trust them. Negative perceptions and experiences of disrespect and belittlement from providers contribute to a lack of trust and embarrassment, making it harder for men to seek medical care (Ab Rahman et al. 2011; Jeffries & Grogan 2012).

The availability and accessibility of healthcare services can also affect decision-making. Our findings demonstrate that men expressed concerns about convenience factors that discouraged them from seeking healthcare. These include long queues and wait times at clinics, which can be frustrating and lead to reluctance to visit healthcare facilities. Some even reported opting for traditional remedies instead of seeking more immediate solutions because of these barriers. The lack of male healthcare professionals in certain areas or specialties may lead to longer waiting times or limited appointment options. This may discourage men from accessing services if they perceive them as inconvenient and time-consuming. In agreement with our findings, a study conducted in New York and Toronto found that protracted waiting times constitute systematic barriers to access healthcare for men (Tudiver & Talbot 1999). Another study also described waiting rooms as being female-oriented, thus making it uncomfortable and inconvenient for men to stay in and wait (Jeffries & Grogan 2012). However, the advent of telehealth and after-hours services has been shown to tackle this context-specific challenge of long waiting times in some settings and should be considered and incorporated into health services (James et al. 2021).

From our study, we also found that having an intimate partner may affect a man’s decision to seek health care, as well as the fear of discovering certain terminal disease conditions that result from poor health education. Female respondents stated that their male partners refused to visit or seek care from health centres because they shared their medicines. While this finding may be true, other studies have shown that having an intimate partner was a facilitating factor for men to seek care when they were sick because of the influence of their partners (Tudiver & Talbot 1999). Thus, having a partner may play a beneficial role or act as a limiting factor in health-seeking behaviours among men. The fear experienced by most men regarding the discovery of certain diseases can be attributed to inadequate health education. When individuals lack sufficient knowledge about specific diseases, they may not fully understand the importance of early detection, appropriate management and lifestyle modifications. This lack of awareness can lead to anxiety and avoidance, which, in turn, hinders their ability to improve outcomes and enhance their overall quality of life. Research findings support our observations, indicating that the primary concerns among men include a lack of knowledge regarding health promotion and preventive measures, fear of illness, and a tendency to deny or ignore symptoms (Barbosa et al. 2018; Tudiver & Talbot 1999).

To address these concerns, efforts can be made to improve gender diversity within the health workforce. Encouraging men to pursue careers in healthcare, particularly in fields where men might prefer male providers, can help create a more balanced workforce. Additionally, promoting cultural competence training for healthcare providers to understand and address men’s unique healthcare needs can enhance the quality of care provided to male patients.

### Potential limitations

This study has a few limitations to consider when interpreting its findings. The sample size was small, so the findings may not apply or be generalised to a larger population without caution. The participants may not represent the full range of experiences of men in the Sedibeng district. There could be a bias in how respondents filled out the questionnaires, potentially affecting the quality of the data collected. Participants may have provided answers that they thought were socially acceptable rather than their true experiences. Another notable limitation was that flyers were accessible only to a limited demographic of educated participants who had access to social media and the internet. Furthermore, participants’ recollection of past experiences may have been imperfect, introducing recall bias. This study did not use data triangulation, which could have strengthened the findings. Future research should address these limitations for a more comprehensive understanding of barriers to healthcare services among men.

### Recommendation and implications of the findings

Based on the findings and insights gained through the ‘Empathise’ and ‘Define’ phases of this study, it is recommended that future research and interventions should prioritise engaging men in the Sedibeng district with innovative and community-driven approaches. The utilisation of online platforms like Facebook, WhatsApp, and the formation of diverse panels involving civil society representatives are essential strategies for co-creating effective programmes aimed at improving men’s participation in healthcare services and addressing gender-based violence in this community.

To address these barriers, it is crucial to implement comprehensive health education programmes that target men, raise awareness about preventive measures, and emphasise the importance of early detection. Efforts should be made to create a supportive and welcoming environment in healthcare facilities by promoting cultural sensitivity, training healthcare providers to be respectful and patient, and ensuring a diverse workforce including male practitioners. Strategies to improve privacy, confidentiality and trust should be implemented to enhance patient–provider relationships. Initiatives such as telehealth and afterhours services can help address convenience factors and reduce long wait times. Collaborative efforts between healthcare providers, community leaders and policymakers are necessary to address these barriers and improve access to healthcare services for men in the Sedibeng district and similar settings.

Overall, by addressing these barriers, healthcare services can become more inclusive, supportive and responsive to the unique needs and concerns of men, ultimately leading to improved health outcomes and a better quality of life.

## Conclusion

This study highlighted several barriers to the uptake of healthcare services among men in the Sedibeng district of South Africa. These barriers include sociocultural factors, stigma and discrimination, demography of health workers, cost of healthcare, use of traditional medicines, convenience, lack of health education, fear of disease outcomes, having an intimate partner, and internal mental models of men. Sociocultural norms contribute to the perception of seeking medical care as a sign of weakness, leading to the denial and hiding of symptoms.

The demography of health workers, particularly the lack of representation of male practitioners, hampers their comfort and trust in discussing sensitive health issues. Negative experiences with female health practitioners contribute to stigma and discrimination, making clinics unwelcome to men. Privacy, confidentiality, and trust also play significant roles in men’s engagement with health care services. The availability and accessibility of services, including long wait times and inconvenient clinical experiences, also discourage men from seeking care. Inadequate health education leads to fear and denial of certain diseases. In conclusion, the initial phases of this study, rooted in community dialogues and human-centred design, have laid a robust foundation for developing targeted and empathetic solutions to enhance men’s engagement with healthcare services in the Sedibeng district.
